# Supplementary material for: Astrocytic p38α MAPK drives NMDA receptor-dependent long-term depression and modulates long-term memory
Source: Nat Commun. 2019 Jul 4;10:2968. doi: 10.1038/s41467-019-10830-9 (PMC6609681; doi:10.1038/s41467-019-10830-9)
Supplement: Supplementary file 1 — Supplementary Information [file 41467_2019_10830_MOESM1_ESM.pdf]

"ASTROCYTIC p38α MAPK DRIVES NMDA RECEPTOR-DEPENDENT LONG-TERM DEPRESSION AND MODULATES LONG-TERM MEMORY"

Navarrete et al

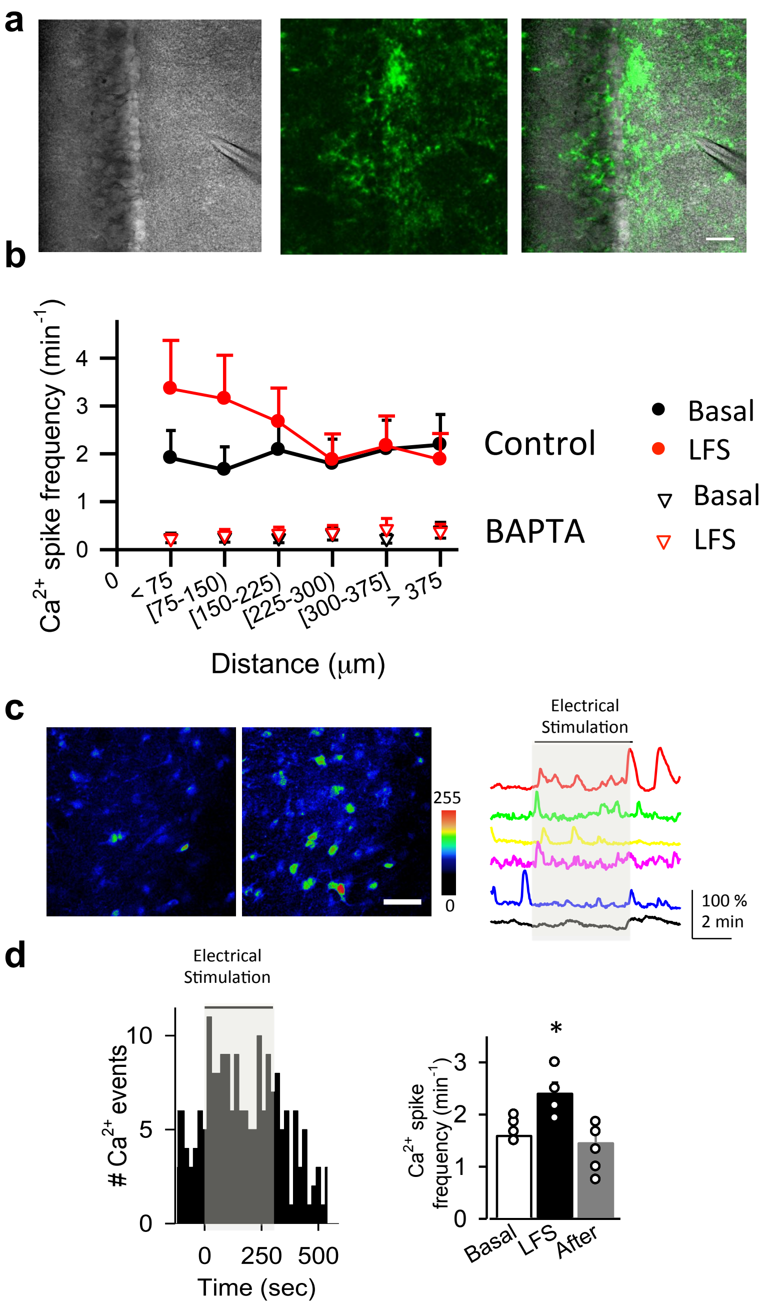

Supplementary Fig. 1. Astrocytes respond with intracellular calcium elevation to LFS.

a Left, Infrared DIC image showing the stimulation electrode and the interest area. Middle, representative fluorescence image showing of a hippocampal acute slice expressing GCaMP6f under GFAP promoter. Right, merge DIC and fluorescence image showing the location of the stimulation electrode and the astrocyte network. Scale bar 40 mm.

b. Ca^{2+} spike frequency versus the respective distance between the ROI and stimulation electrode tip in control acute hippocampal slices (circles) and in acute slices with BAPTA-filled astrocytes (triangles). Both basal spike frequency (black symbols) and during LFS (red symbols) are plotted.

c Representative pseudocolor images (left) and quantification (right) of calcium signals from six astrocytes, obtained from fluorescence intensities of fluo-4-filled astrocytes in hippocampal acute slices before, during and after LFS protocol (300 pulses at 1 Hz). Scale bar, 50 µm.

d Left, Representative experiment showing the number of calcium events during a LFS experiment in time bins of 20 seconds. Right, average frequency of Ca^{2+} signals per astrocyte before (baseline), during (LFS) and after LFS (n = 79 astrocytes from 6 slices). Data were compared by non-parametric Friedman test followed by Dunn´s multiple comparison (*P < 0.05).

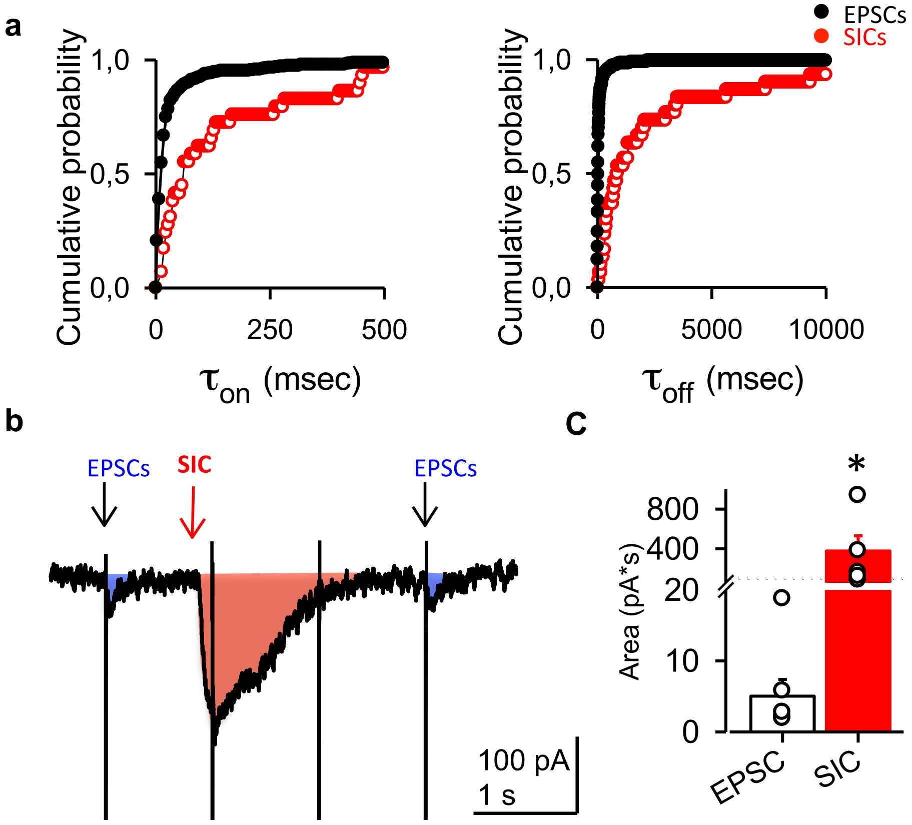

Supplementary Fig. 2. Kinetic analysis of EPSC and SIC events during LFS.

a Cumulative distribution of onset (left) and offset (right) times from individual EPSCs and SICs.

b Representative trace from a LFS experiment carried out without Mg^{2+} to visualize NMDA receptor-mediated EPSCs (blue) and SICs (red). Shadowed areas represent total charge transfer from each event. Stimulation artifacts at 1 Hz are visible as vertical lines.

c Average charge transfer from EPSCs and SICs, calculated from 7 different experiments, as the one shown in B. Data were compared by Mann-Whitney test (* $P < 0.05$).

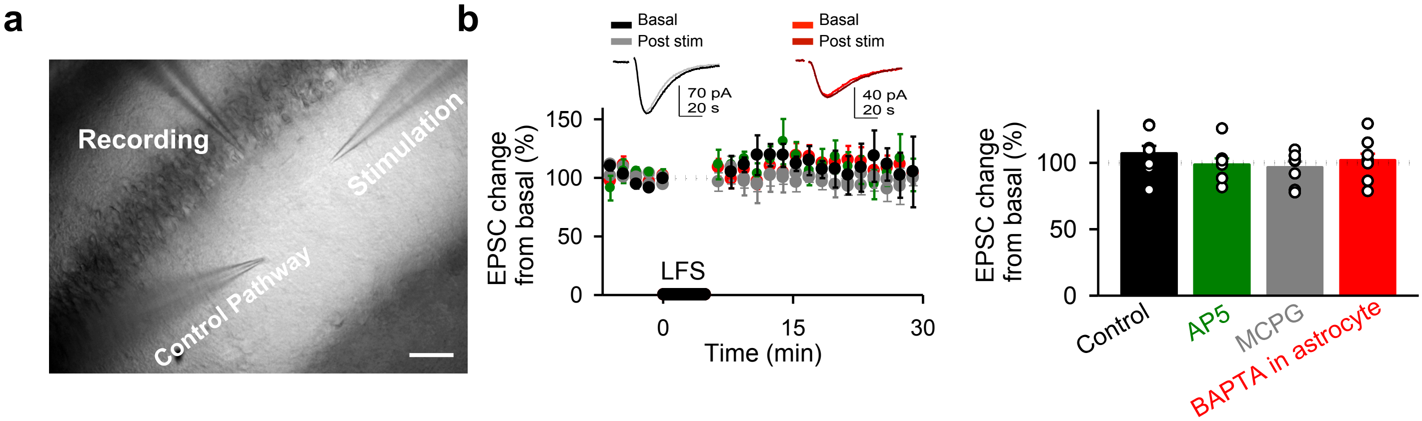

Supplementary Fig. 3. LFS-induction of NMDAR-dependent homosynaptic depression.
a Infrared differential interference contrast (DIC) image showing recorded CA1 pyramidal neuron and the stimulation electrodes in an acute hippocampal slice. Scale bar, 50 µm.
b EPSC change from basal values *versus* time from the stimulating electrode that was turned off during LFS protocol (“control pathway”). Data are plotted for control acute slices, AP5, MCPG and BAPTA treatments, corresponding to Fig. 1 of the main text. Differences between groups were determined by Kruskal-Wallis test followed Dunn´s test. Wilcoxon statistical test was used to analyze LTD expression with respect to baseline.

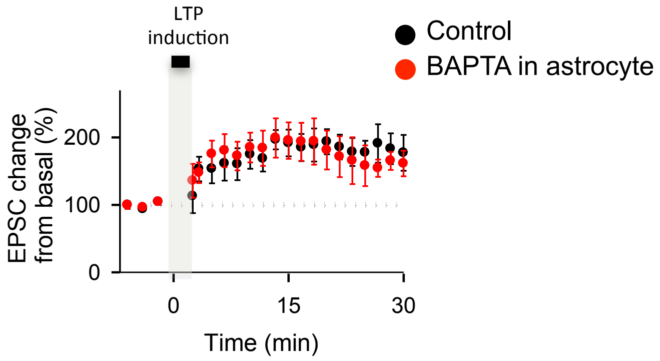

Supplementary Fig. 4. Astrocytic BAPTA infusion does not alter LTP.

Relative EPSC amplitudes before and after LTP induction (300 pulses at 3Hz, 0 mV postsynaptic depolarization) in control (black, n = 8) and BAPTA-loaded astrocytes (red, n = 7) from acute hippocampal slices. Zero time correspond to the onset of the LTP protocol. Mann-Whitney *U*-test; P = 0.46.

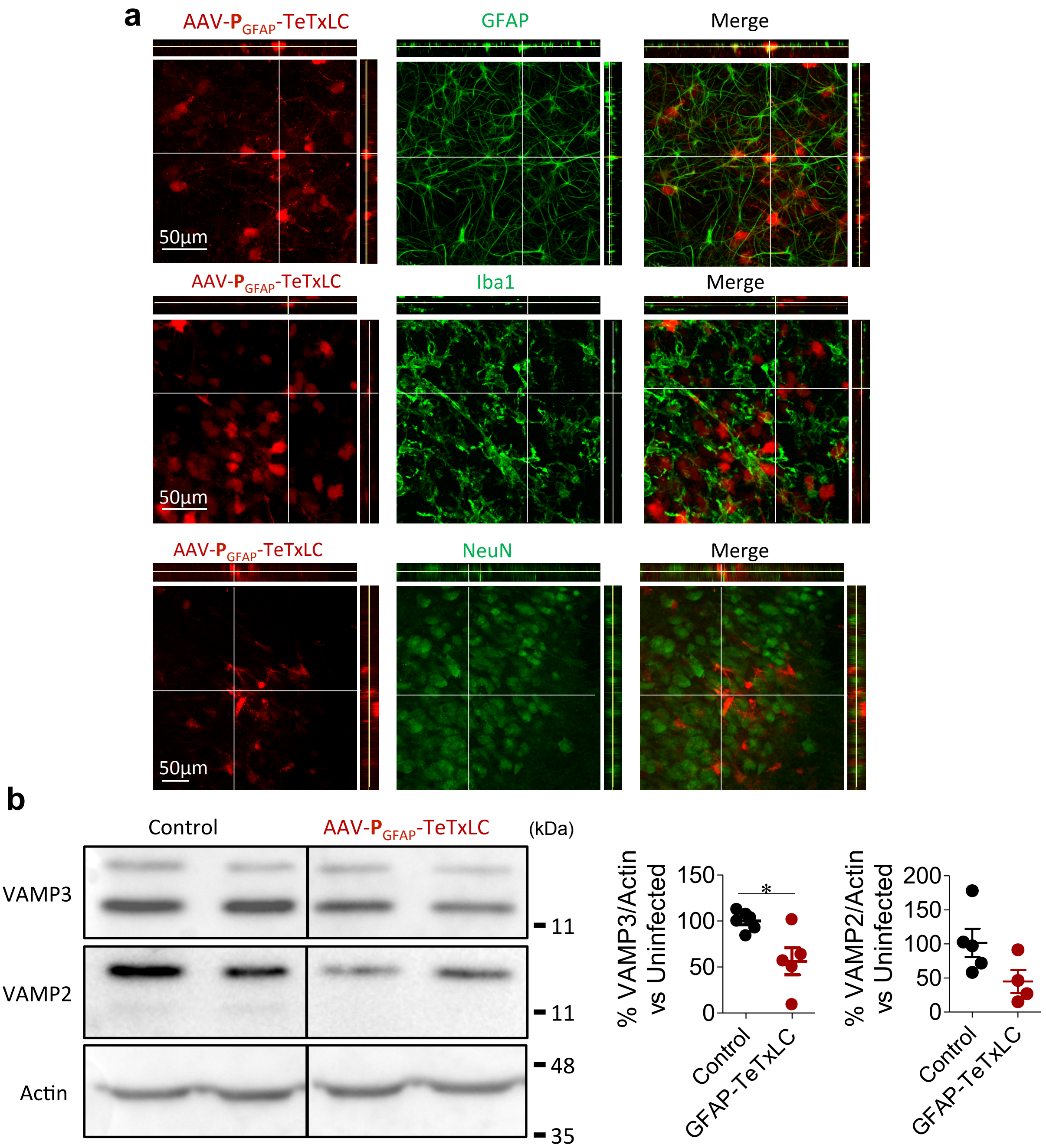

Supplementary Fig. 5. Viral expression of TeTxLC selectively in astrocytes.
a Immunohistochemical analysis of TeTxLC colocalization in organotypic hippocampal slices with astrocytic (GFAP), microglial (Iba1) and neuronal (NeuN) markers. Scale bars, 50 µm. Orthogonal projections from stack images are shown.
b Western blot examples (left) and quantification (right) of VAMP3 (cellubrevin) and VAMP2 (synaptobrevin2) protein levels from uninfected (Control) organotypic hippocampal slices (n=5-6) and from infected slices expressing $\mathbf{P}_{GFAP}$-TeTxLC (n=4-5). Actin served as a loading control. Data are displayed as the % of uninfected slices. Data were compared by nonparametric Mann Whitney test (* P < 0.05).

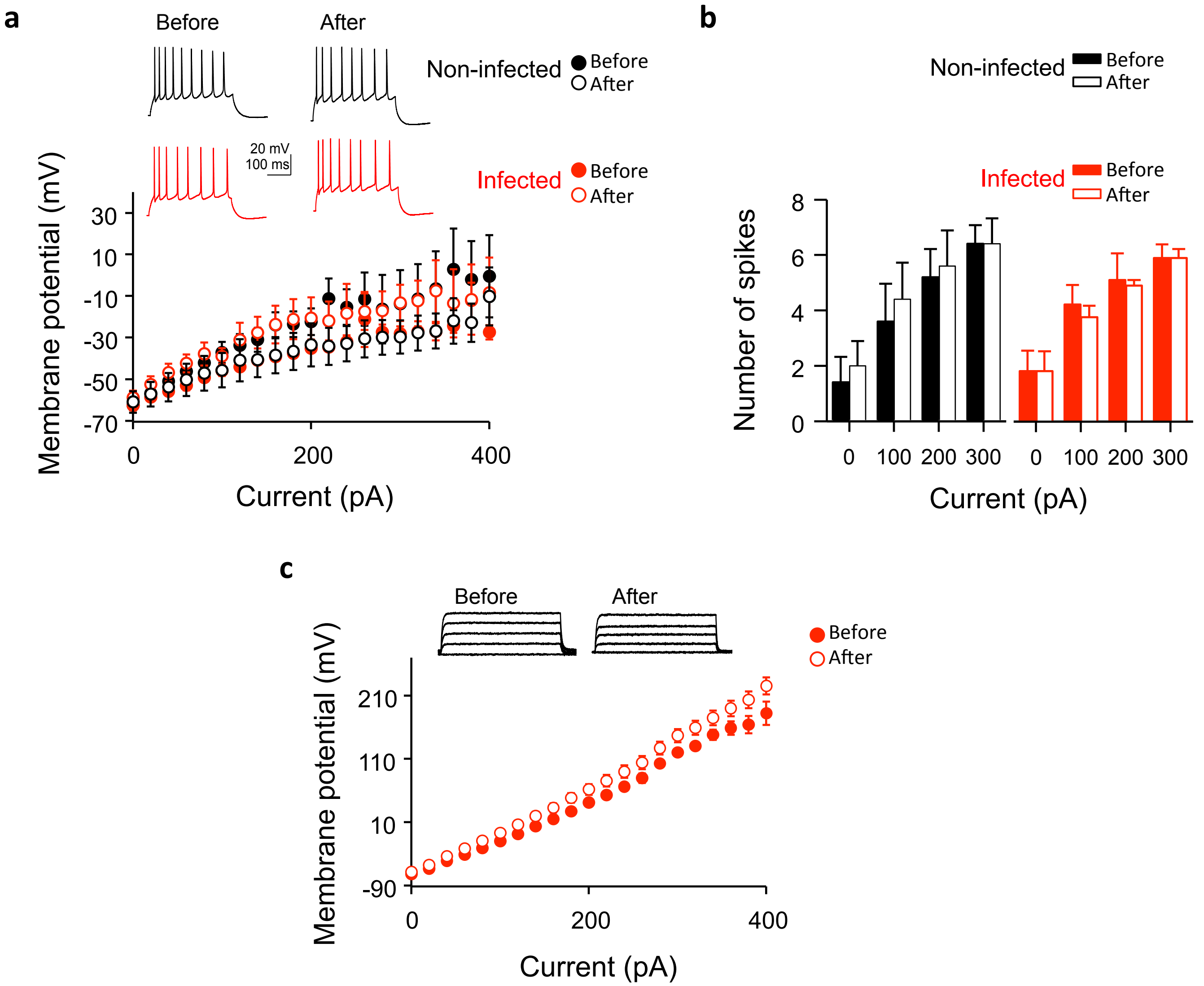

Supplementary Fig. 6. Neuronal and astrocytic electrical properties.

a (Top) Representative recordings of changes in membrane potential and action potential firing of CA1 neurons upon current injection, from organotypic slices before and after optogenetic stimulation, with or without ChR2-expression in astrocytes, as indicated. (Bottom) Average changes in neuronal membrane potential upon different current injections, from recordings as the one shown at the top. n = 7 and n = 5 neurons in control and ChR2-expressing slices respectively.

b Average number of action potentials fired at different current injections, from recordings as the one shown in A. n = 7 and n = 5 neurons in control and ChR2-expressing slices respectively.

c (Top) Representative recordings of changes in membrane potential of ChR2-expressing astrocytes upon current injection, from organotypic slices before and after optogenetic stimulation. (Bottom) Average changes in astrocytic membrane potential upon different current injections, from recordings as the one shown at the top. n = 5 astrocytes for both conditions.

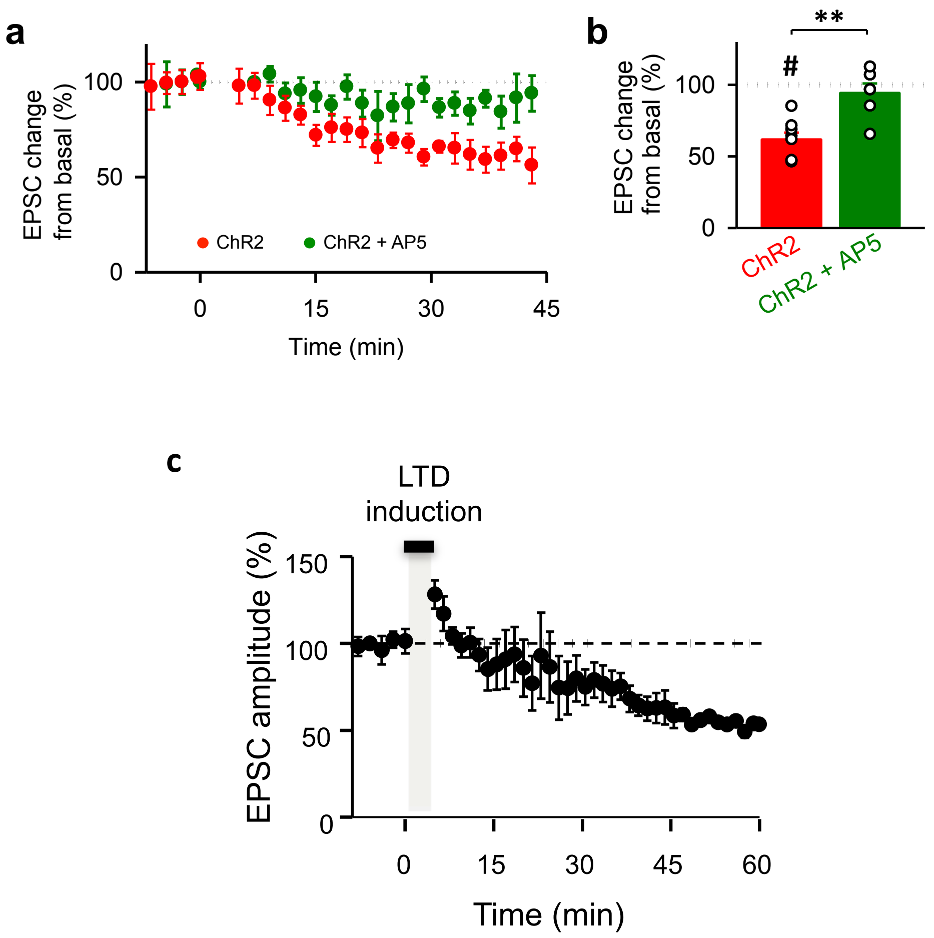

Supplementary Fig. 7. Optogenetic LFS in acute slices. Control recordings without MCPG.
a Time course of CA3-CA1 EPSC amplitudes during a photostimulation experiment (gray bar) from control Ch2R-expressing acute hippocampal slices (red, n = 8) and in presence of the NMDAR antagonist AP5 (green, n = 6)
b Average values of EPSC amplitude relative to baseline, 45 min after the onset of photostimulation. Data were compared by nonparametric Mann Whitney test (** P < 0.01). Data are presented as means ± s.e.m.
c Relative and average EPSC amplitudes from ChR2-expressing organotypic hippocampal slices before and after photostimulation (300 pulses at 1Hz, -40 mV postsynaptic depolarization) without MCPG in the bath (n = 4). Zero time correspond to the onset of the LTD protocol. Wilcoxon test, p < 0.05, comparing the end of the time course with baseline transmission.

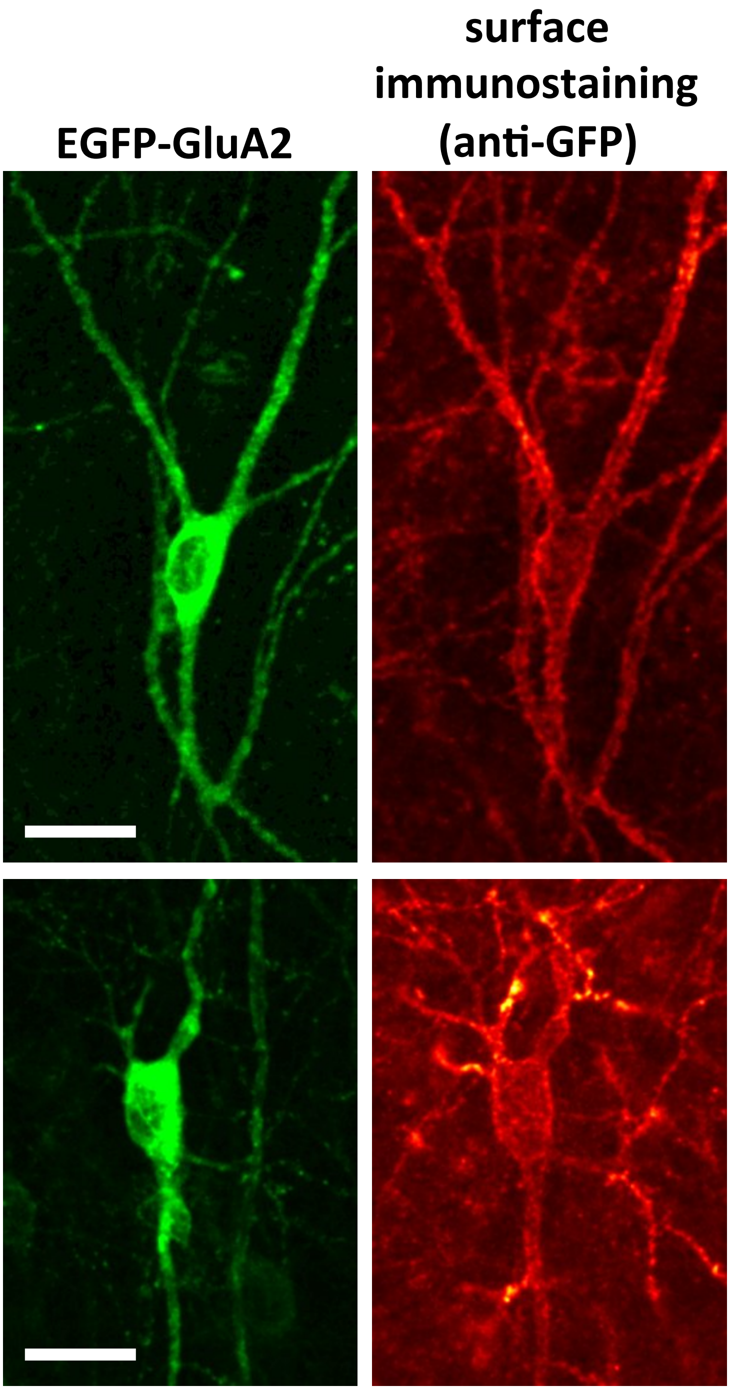

Supplementary Fig. 8. Surface immunostaining in organotypic hippocampal slices.
Representative confocal images of CA1 neurons expressing EGFP-GluA2 in organotypic hippocampal slices. Green channel (left) represents total receptor expression (EGFP signal) and red signal (right) represents surface expression (anti-GFP immunostaining under non-permeabilizing conditions). Note membrane-delimited signal for surface immunostaining, as compared to whole-cell (nucleus excluded) fluorescence of EGFP-GluA2. Scale bars, 20 μm.

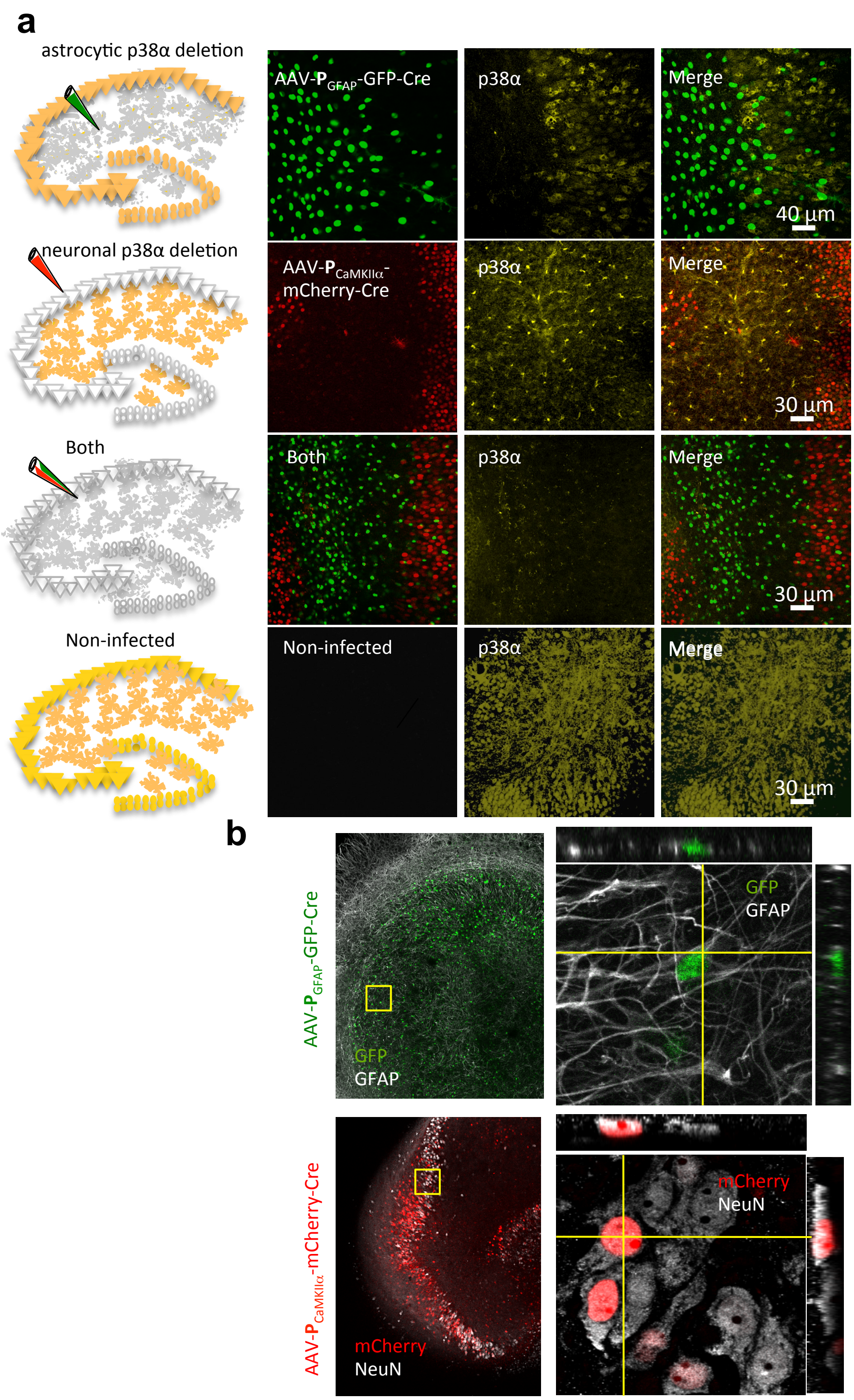

Supplementary Fig. 9. Cell type-specific p38α removal by AAV-driven Cre expression.

a (Left) Experimental strategy for p38α genetic deletion in astrocytes, neurons, or both cell types in organotypic hippocampal slices from p38α$^{lox/lox}$ mice. (Right) immunohistochemical analysis of p38α expression (yellow channel) from the experimental configurations described on the left. Green channel: GFP-Cre expression under the GFAP promoter. Red channel: mCherry-Cre expression under the CaMKIIα promoter. Scale bars, 40 (top) and 30 µm.

b Immunohistochemical analysis of Cre colocalization in **P**$_{GFAP}$-Cre (upper panels) or **P**$_{CaMKIIα}$-Cre-infected (lower panels) p38α$^{lox/lox}$ organotypic hippocampal slices with astrocytic (GFAP) and neuronal (NeuN) markers. Orthogonal projections from stack images are shown.

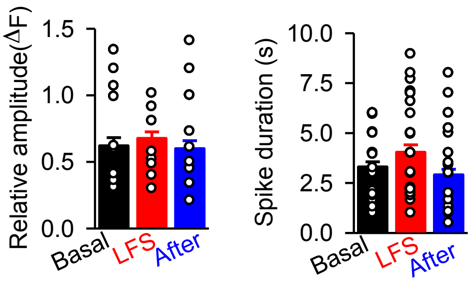

Supplementary Fig. 10. Kinetic analysis of astrocytic iGluSnFR spikes.

Relative amplitude (left) and duration (right) of glutamate spike fluorescent signals before, during and after the LFS protocol (binning of 5 min) in organotypic hippocampal slices expressing iGluSnFR in astrocytes.

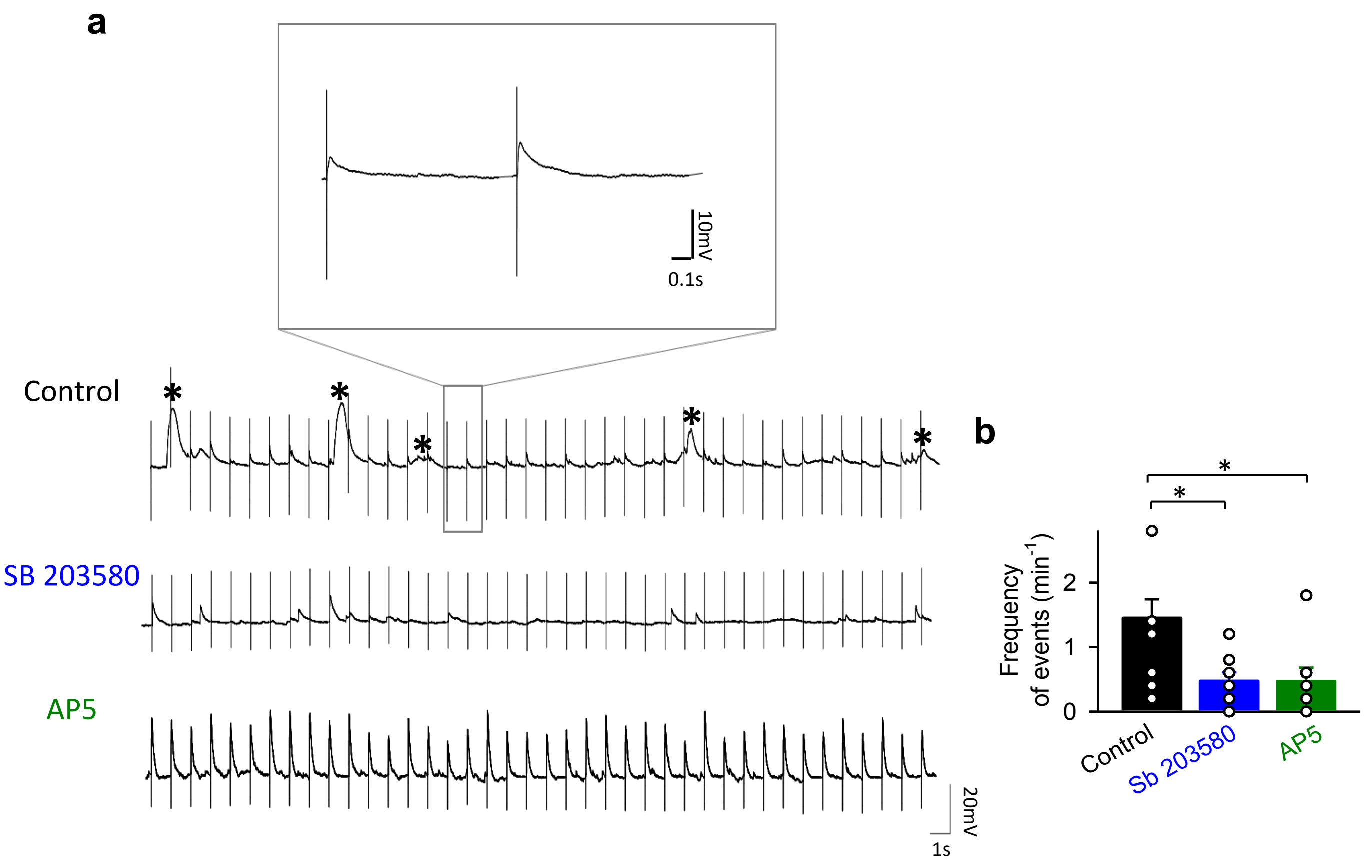

Supplementary Fig. 11. Membrane depolarization from CA1 neurons during LFS protocol.
a Representative current-clamp recordings of CA1 pyramidal neurons loaded with QX-314 during LFS stimulation of Schaffer collaterals (1 Hz, 300 pulses) in acute hippocampal slices. Basal resting membrane potential is set to around −40 mV by small current injections. Upper panel: control conditions. Middle panel, in the presence of the p38α/p38b inhibitor SB203580 (5 mM). Lower panel, in presence of the NMDAR antagonist AP5 (50 μM). Inset, zoom-in of postsynaptic responses to LFS stimulation under control conditions.
b Frequency of slow membrane depolarizations (mean area = 2441 ± 555 mV*ms, rise time 940 ± 22.5 ms, n = 68 events) in control conditions (black, n = 8), in presence of p38α/p38b inhibitor SB203580 (blue, n = 8) and in presence of the NMDAR antagonist AP5 (green, n = 8). Turkey post-hoc test after one-way ANOVA, $P < 0.05$.

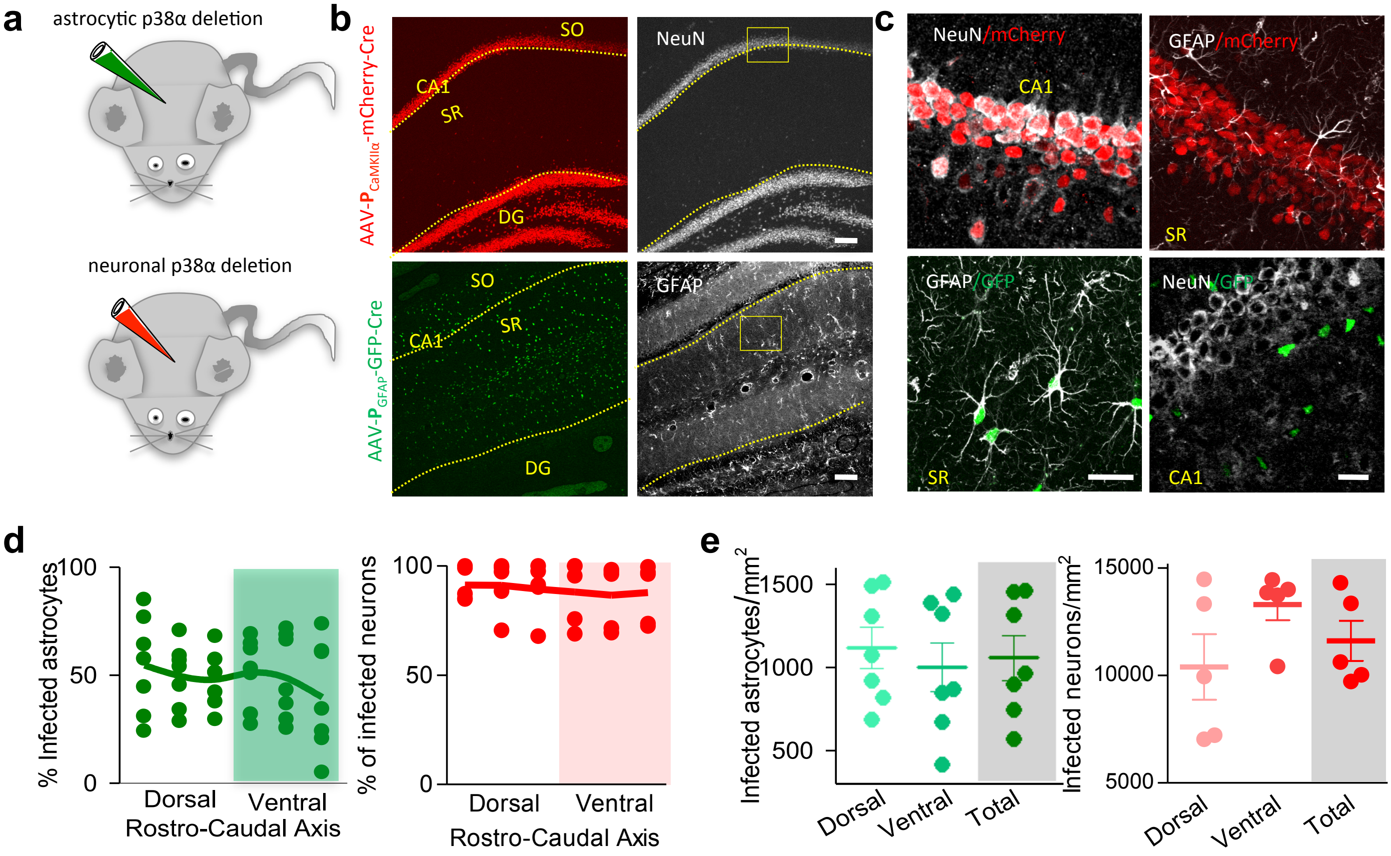

Supplementary Fig. 12. *In vivo* cell type-specific deletion of p38α with AAV.

a Schematic drawing showing the experimental approach for p38α genetic deletion in hippocampal astrocytes or neurons by *in vivo* injection in p38a$^{lox/lox}$ mice by *in vivo* injection in p38a$^{lox/lox}$ mice.

b Colocalization analysis of Cre with NeuN and GFAP in brain sections from p38α$^{lox/lox}$ mice determined by immunohistochemistry 30 days after infection with AAV5-**P**$_{CaMKII\alpha}$-mCherry-Cre (top panel) and AAV5-**P**$_{GFAP}$-GFP-Cre (bottom panel). Scale bars, 100 µm. SO (*stratum oriens*), SR (*stratum radiatum*), DG (d*entate gyrus*).

c High magnification images from the square regions indicated in (B). Scale bars, 10 µm.

d Percentage of infected astrocytes (GFP/GFAP positive cells; n = 7 mice) in the *stratum radiatum (left)* and (mCherry/NeuN positive cells, n = 5 mice) in the *stratum pyramidale* (right) of infected p38α$^{lox/lox}$ mice determined along the rostro-caudal hippocampal axis. Data from individual mice are displayed as the average of infection levels found in both right and left hippocampus.

e Quantification of the numbers of infected astrocytes (green graph; n = 7 mice) and neurons (red graph; n = 5 mice) per mm^2 determined for dorsal, ventral and total hippocampus. Data from individual mice were calculated as the average from both right and left hippocampus. Data are presented as means ± s.e.m.

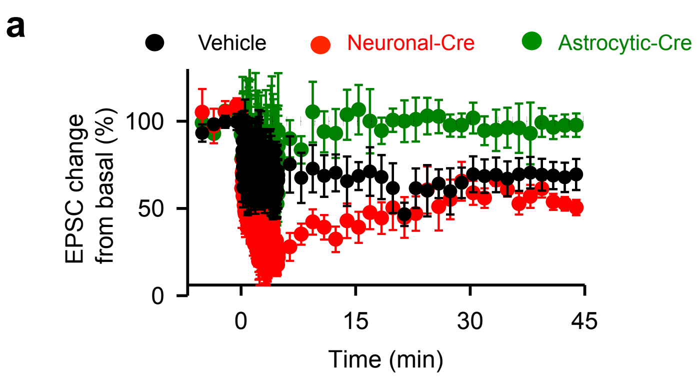

Supplementary Fig. 13. Impaired LTD in acute slices with astrocytic deletion of p38a.
a Relative EPSC amplitudes (normalized to baseline values) *versus* time and **b** average relative changes of EPSC amplitudes 45 min after electrical LFS in acute hippocampal slices from p38α$^{lox/lox}$ animals 1 month after being infected with $\mathbf{P}_{CaMKII\alpha}$-Cre (red, n = 11 from n = 6 mice), $\mathbf{P}_{GFAP}$-Cre (green, n = 11 from n = 7 mice) and vehicle-treated (black, n = 8 from n = 6 mice). Bonferroni post-hoc test after one-way ANOVA ($F\ (2, 29) = 11.63$; $p < 0.001$). Wilcoxon statistical test was used to analyze LTD expression with respect to baseline. Data are presented as means ± s.e.m.

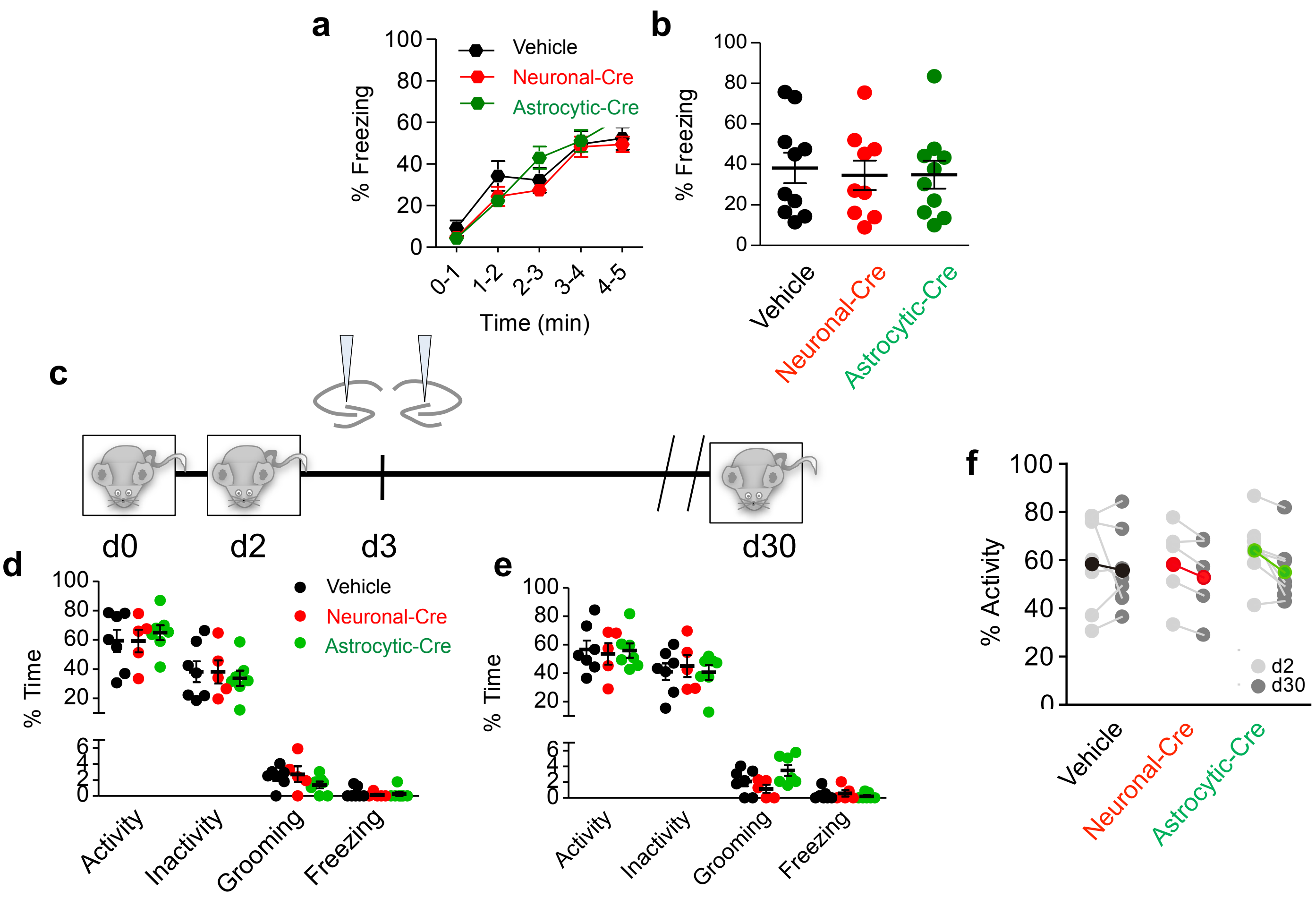

Supplementary Fig. 14. Behavioral controls of p38α$^{lox/lox}$ mice. a Freezing levels observed in p38α$^{lox/lox}$ during the training phase of contextual fear conditioning. Two-way ANOVA: F(4; 116)=79.8, p<0.001. Control p38α$^{lox/lox}$ (vehicle, N=11 mice), neuronal Cre (N=10 mice) and astrocytic Cre (N=11 mice). **b** Percentage of freezing observed 2 days after contextual fear conditioning (pre-infection test, d2) in p38α$^{lox/lox}$ mice before infection. Control p38α$^{lox/lox}$ (vehicle, N=1 mice), neuronal Cre (N=10 mice) and astrocytic Cre (N=11 mice). Kruskal-Wallis statistic =0.19; p=0.91. **c** Cartoon representing the protocol used for non-shocked p38α$^{lox/lox}$ mice. On day 0, p38α$^{lox/lox}$ mice were trained similarly to mice of Figure 6, but they did not receive the foot shock. Two days later, mice were briefly tested (3 minute re-exposure; pre-infection test; d2) to check mice behavior before infection. The next day (d3), mice were allocated in one of the three experimental groups: saline or either AAV-expressing Cre under CaMKIIα (neuronal Cre) or under GFAP promoter (astrocytic-Cre) was bilaterally delivered in both dorsal and ventral hippocampi. 30 days after training, infected mice were tested for different behaviors including activity, grooming, inactivity and freezing (post-infection test, d30). **d** Percentage of freezing observed in non-shocked p38α$^{lox/lox}$ control mice (vehicle-treated) and neuronal-Cre (red symbols) and astrocytic-Cre groups (green symbols) during the pre-infection test 2 days after training. Graph bars also display the percentage of time spent by p38α$^{lox/lox}$ in different behaviors such as activity, inactivity and grooming. Two-way ANOVA demonstrated no significant differences between experimental groups (F(6; 64)=0.27, p=0.95). Control p38α$^{lox/lox}$ (n=7 mice), neuronal Cre (n=5 mice) and astrocytic Cre (n=7 mice). **e** Similar to **(d)**, but tested on animals 30 days after training (post-infection test). Two-way ANOVA demonstrated no significant differences between experimental groups (F(6; 64)=0.15, p=0.99). Control p38α$^{lox/lox}$ (n=7 mice), neuronal Cre (n=5 mice) and astrocytic Cre (n=7 mice).**f** Comparison of activity levels (percentage of time spent moving) displayed by each animal and group at the pre-infection (d2) and post-infection tests (d30). No significant differences were detected by Two-way ANOVA (F(2; 16)=0.60, p=0.56). Control p38α$^{lox/lox}$ (n=7 mice), neuronal Cre (n=5 mice) and astrocytic Cre (n=7 mice).

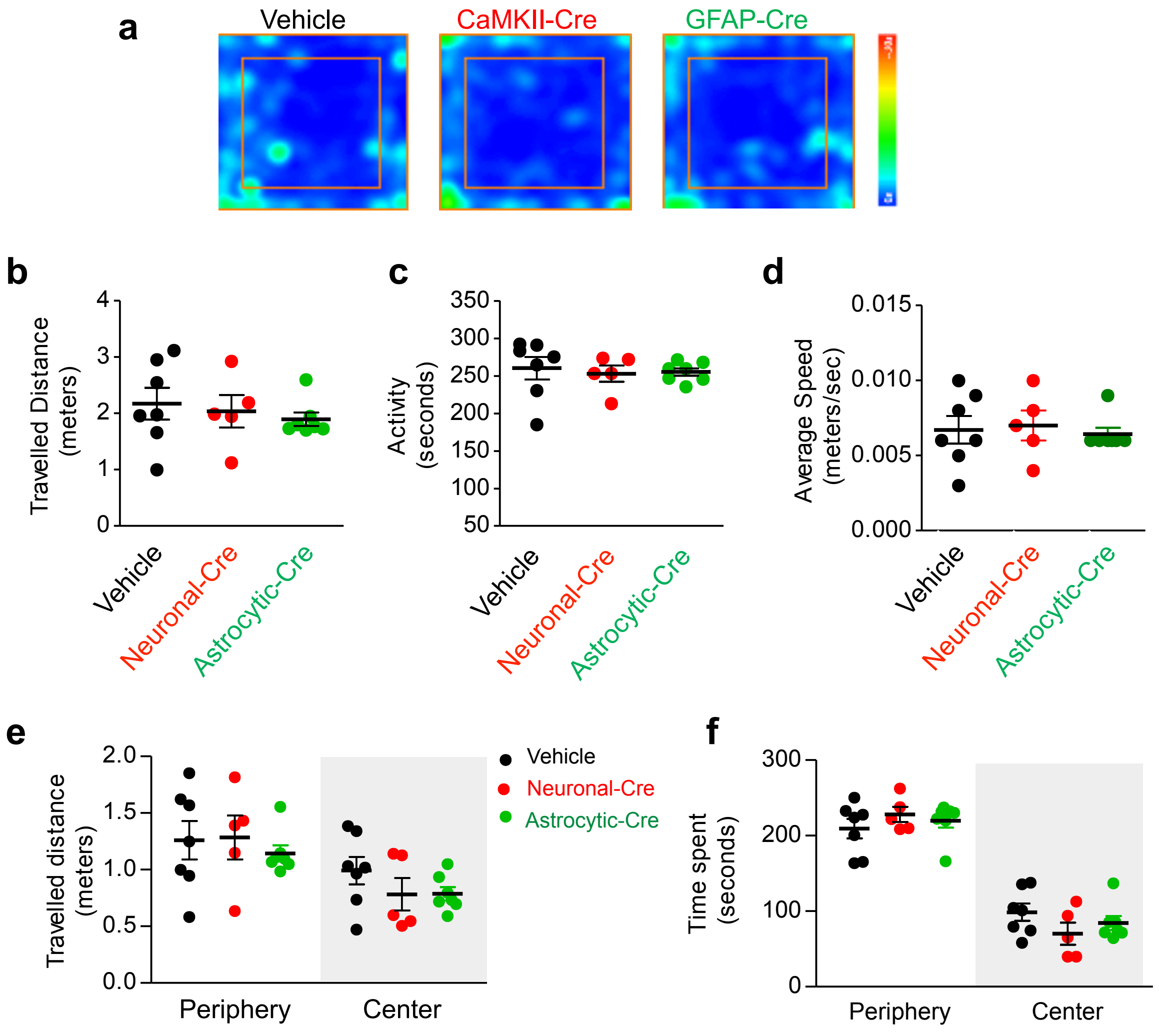

Supplementary Fig. 15. Open field analysis of p38α $^{lox/lox}$ mice.

a Representative heat maps of locomotor activity observed in p38α$^{lox/lox}$ control mice and p38α$^{lox/lox}$ infected either with neuronal-Cre (red symbols) or astrocytic-Cre virus (green symbols).

b-d Open field analysis plotting distance travelled by each group (b), the mobility time in seconds (c) and the mean speed (d) over a 5 min session. Control p38α$^{lox/lox}$ (n=7 mice), neuronal Cre (n= 5 mice) and astrocytic Cre (n=7 mice). Data were compared by non-parametric Kruskall-Wallis test.

e-f Travelled distance (e) and time spent (f) in the center or periphery by p38α$^{lox/lox}$ control mice (vehicle-treated) and p38α$^{lox/lox}$ infected either with neuronal-Cre (red symbols) or astrocytic-Cre virus (green symbols) 30 days after infection. Control p38α$^{lox/lox}$ (n=7 mice), neuronal Cre (n= 5) and astrocytic Cre (n=7 mice). No differences were observed in either (e) or (f) by Two Way ANOVA analysis.

Figure	Ns<br>n refers to slices N refers to animals	Statistical Analysis	
1c (calcium spike)	Control (n = 20; N = 6)	**Friedman test**; p < 0.0001<br>Friedman statistic = 27,70.<br>Dunn´s Multiple comparisons<br>Before vs LFS<br>Before vs After<br>LFS vs After	Difference in rank sum<br>´-26,00; p < 0.001<br>5,000;p > 0.05<br>31,00; p < 0.001
1d (SICs)	Control (n = 12; N = 8)<br>AP5 (n = 8; N = 6)	**Friedman test**; p = 0.0009<br>Friedman statistic = 12,07.<br>Dunn´s Multiple comparisons // Difference in rank sum<br>Before vs LFS; ´-13; p < 0.01<br>Before vs After; ´-3.5; ns<br>LFS vs After; ´9.5; ns	**Mann-Whitney U-test**<br>Before vs AP5 < 0.001, z = 3.664
2b (LTD)	Control (n = 10; N =6)<br>AP5 (n = 8; N = 7)<br>MCPG (n = 8, N = 5)<br>BAPTA (n = 12, N = 8)<br>IP3R2$^{-/-}$ (n = 13, N = 7)	**Kruskal-Wallis test**; p = 0.002<br>Kruskal-Wallis statistic = 22,36<br>Dunn´s Multiple comparisons // Difference in rank sum<br>Control vs Bapta, p < 0.01 ; -21,42<br>Control vs AP5, p < 0.01; -25,50<br>Control vs IPR3-/-, p < 0.05 ; -19,45	**Wilcoxon signed-rank test**<br>Control: p < 0.01; z = 2.77<br>AP5: p = 0.81; z = 0.24<br>MCPG: p < 0.05; z = 2.48<br>BAPTA: p = 0.06; z = 1.9<br>IP3R2$^{-/-}$: p = 0.06; z = 1.9
2d (SICs, TeTxLC)	Control (n = 4, N = 3)<br>TeTxLC (n = 5, N = 4)	**Two-way ANOVA :**<br>Infection; F(1;12) = 51.08, p= 0,0004<br>Bonferroni post-hoc<br>Control vs Astrocytic TeTxLc<br>Baseline; p > 0.05 (t=2,407)<br>LTD; p < 0,001 (t=5,341)<br>After; p < 0.01 (t=3,616)	**Friedman test control; p = 0.0009**<br>Friedman statistic =12,07.<br>Dunn´s Multiple comparisons / Difference in rank sum<br>Before vs LFS; ´-13; p < 0.01<br>Before vs After; ´-3.5; ns<br>LFS vs After; ´9.5; ns<br>**Friedman test TeTxLc; p = 0.6914**<br>Friedman statistic = 0.5<br>Dunn´s Multiple comparisons / Difference in rank sum<br>Before vs LFS; 1.5; ns<br>Before vs After; 1.5; ns<br>LFS vs After; 0; ns
2e (TeTxLC)	Control (n = 5, N = 3)<br>TeTxLC (n = 7, N = 4)		**Wilcoxon signed-rank test**<br>Control: p < 0.05; z = 2.14<br>TeTxLC: p = 0.07; z = 1.82
3c (Frequency Ca2+)	n = 300 astrocytes n = 11, N = 5	**Friedman test**; p < 0.0001<br>Friedman statistic = 16.91.<br>Dunn´s Multiple comparisons ///// Difference in rank sum<br>Before vs LFS; ´-18; p < 0.001<br>Before vs After; ´-3; ns<br>LFS vs After; ´15; np < 0.01	
3c (Relative Amplitude)	n = 69 astrocytes n = 9, N = 5	**Friedman test**; p = 0.0002<br>Friedman statistic = 13.94<br>Dunn´s Multiple comparisons ///// Difference in rank sum<br>Before vs LFS; ´-10.50; p < 0.05<br>Before vs After; 4.5; ns<br>LFS vs After; ´15; p < 0.01	
3d (Sics frequency)	n = 13, N = 5	**Friedman test**; p = 0.0174<br>Friedman statistic = 7,548<br>Dunn´s Multiple comparisons ///// Difference in rank sum<br>Before vs LFS; ´-10.50; p<0.05<br>Before vs After; 3; ns<br>LFS vs After; 7.5; ns	
4a (chr2)	Non infected (n = 7, N = 5)<br>Infected (n = 8, N = 4)<br>AP5 (n = 8, N = 5)<br>Ifenprodil (n = 6, N = 3)	**Kruskal-Wallis test**; p = 0.0044<br>Kruskal-Wallis statistic = 13.09<br>Dunn´s Multiple comparisons ///// Difference in rank sum<br>Control vs infected, p < 0.05 ; 12.98<br>Infectedl vs AP5, p < 0.01; -13.79<br>Infected vs ifenprodil, p < 0.05 ; -10.79	**Wilcoxon signed-rank test**<br>Non infected: p = 0.21; z = 1.21<br>Infected: p < 0.05; z = 2.32<br>AP5: p = 0.22; z = 1.22<br>Ifenprodil: p = 0.08; z = 1.73
4b (desp)	Cocktail (n = 8, N = 4)<br>D-Serine (n = 6, N = 4)<br>No postsynaptic depolarization (n = 7, N = 3)<br>0 Mg2+ (n = 7, N = 4)	**Kruskal-Wallis test**; p = 0.0013<br>Kruskal-Wallis statistic = 15.74<br>Dunn´s Multiple comparisons ///// Difference in rank sum<br>Cocktail vsno post desp, p < 0.05 ; -11.86<br>No post depl vs 0 Mg2+, p < 0.001; 16.86	**Wilcoxon signed-rank test**<br>Coktail: p < 0.05; z = 2.34<br>D-Serine: p < 0.05; z = 2.15<br>No post dep p = 0.09; z = 1.73<br>0 Mg^{2+}: p < 0.01; z = 2.32
5b (MK801)	Control elec stim (n = 6, N = 5)<br>MK801 elec stim (n = 6, N = 5)<br>Control ChR2 stim (n = 7, N = 4)<br>MK801 ChR2 stim (n = 7, N = 4)	**Two-way ANOVA:**<br>F (1, 19) = 0.02, p = 0.90<br>MK801: F (1, 22) = 14.64, p < 0.001	**Wilcoxon signed-rank test**<br>Basal vs LFS: z < 0.05; z 0.90; z < 0.05; z = 0.64<br>Basal vs LFS: z < 0.05; z 0.90; z < 0.05; z = 0.64
5d (AMPAR Removal)	Surface (n > 116 spines n = 3, N = 3)<br>Total (n > 116 spines n = 3, N = 3)<br>t = 0; 362 spines n = 3, N = 3<br>t = 5; 197 spines n = 3, N = 3<br>t = 10; 393 spines n = 3, N = 3<br>t = 35; 116 spines n = 3, N = 3	**One-way ANOVA**<br>F = 20.49<br>Dunnett's Multiple Comparison Test<br>p < 0.001	

6c (p38 mice)	p38 beta-/- (n = 4, N = 4)<br>p38beta -/- + p38 alpha CamKII -/- (n = 4, N = 4)	**Mann-Whitney U-test**<br>Wt vs p38 beta; p < 0.001; z = 2.16<br>Wt vs double p38; p < 0.001; z = 2.16<br>Alpha: Mann-Whitney U-test<br>Wt vs p38 beta; p = 0.31; z = 1.01<br>Wt vs double p38; p < 0.001	**Kruskal-Wallis test**; p=0.0179; statistic=7,584<br>Wt vs p38 beta; p < 0.001;6,756<br>Wt vs double p38; p < 0.001; 5.675<br>**Kruskal-Wallis test**; p=0.0061; 10,20<br>Wt vs p38 beta; p = 0.31; z = 1.01<br>Wt vs double p38; p < 0.001
6d Basal transmission	WT (n = 48, N = 8)<br>p38beta -/- (n = 39, N = 7)<br>p38beta -/- + p38 alpha CamKII -/- (n = 39, N = 8)	**Two-way ANOVA:**<br>F (2, 240); p = 0.7321	
6e (fEPSP) p 38 mice	WT (n = 11, N = 7 e)<br>p38beta-/- (n = 11, N = 7)<br>p38beta -/- + p38 alpha CamKII -/- (n = 9, N = 7)	**Kruskal-Wallis test**; p=0.0179; statistic=7,584<br>Wt vs p38 beta; p < 0.001;6,756<br>Wt vs double p38; p < 0.001; 5.675	**Wilcoxon signed-rank test**<br>WT: p < 0.01; z = 2.91<br>p38beta-/-: p < 0.01; z = 2.64<br>p38beta double -/- p < 0.01; z = 2.63
7b (EPSC)	Non infected (n = 7, N = 4)<br>Non infected + SB203580 (n = 7, N = 4)<br>Neuronal Cre (n = 6, N = 4)<br>Neuronal Cre + SB203580 (n = 6, N = 3)<br>Astrocyte Cre (n = 8, N = 5)	**Kruskal-Wallis test**<br>F (4, 29) = 3.57 , P < 0.05<br>**Mann-Whitney U-test**<br>Non infected vs non infected + sb203580, p < 0.05, z = 2.62<br>Non infected vs neuronal cre, p = 0.93, z = 0.16<br>Non infected vs neuronal cre + sb203580, p = 0.93, z = 0.16<br>Non infected vs neuronal cre + sb203580, p = 0.93, z = 0.16	**Wilcoxon signed-rank test**<br>Non infected: p < 0.05; z = 2.14<br>Non infected + SB203580: p = 0.19; z = 1.31<br>Neuronal Cre: p < 0.05; z = 2.15<br>Neuronal Cre + SB203580: p = 0.27; z = 1.10<br>Astrocyte Cre:p = 0.17; z = 1.32
8e (Glutamate imaging)	Control (n = 7, N = 4)<br>p38 alpha knock out slice (n = 8, N = 4)	**Two-way ANOVA:**<br>interaction;´ F(2;39) = 5,82, p= 0,0425<br>Bonferroni post-hoc<br>Control vs Astrocytic<br>LTD; Difference = -0,7919; t = 2,913;p < 0.05	**Friedman test control; p=0.0009**<br>Friedman statistic =12,07.<br>Dunn´s Multiple comparisons /Difference in rank sum<br>Before vs LFS; ´-13; p<0.01<br>Before vs After; ´-3.5; ns<br>LFS vs After; ´9.5; ns<br>**Friedman test p38; p=0.0009**<br>Friedman statistic =12,07.<br>Dunn´s Multiple comparisons / Difference in rank sum<br>Before vs LFS; ´-13; p< 0.01<br>Before vs After; ´-3.5; ns<br>LFS vs After; ´9.5; ns
8g (SIcs)	Control (n = 14, N = 4)<br>p38 alpha knock out slice (n = 17, N = 6)	**Two-way ANOVA:**<br>Treatment; F(1;87)=5,82, p = 0,0179<br>Bonferroni post-hoc<br>Control vs Astrocytic<br>LTD; Difference=-0,7919; t = 2,784;p < 0.05	
9c	Vehicle (N = 11)<br>Neuronal Cre (N = 9)<br>Astrocyte Cre (N = 11)	**Kruskal-Wallis test**<br>Kruskal-Wallis statistic = 9,843; p = 0,007<br>Dunn's Multiple Comparison Test<br>Vehicle vs GFAP-Cre ; Difference in rank sum = -8,63; p < 0.05<br>Vehicle vs CaMKIIa-Cre: Difference in rank sum=3,827;p > 0.05<br>CaMKIIa-Cre vs GFAP-Cre; Difference in rank sum =-12,46; p < 0.05	
9d	Vehicle (N = 11)<br>Neuronal Cre (N = 9)<br>Astrocyte Cre (N = 11)	**Two-way ANOVA:**<br>Interaction; F(2;57) = 3,286, p = 0,045<br>Bonferroni post-hoc<br>Control vs GFAP-Cre<br>Before; Difference = -3,29; t= 0,3753; p > 0.05<br>After; Difference = 21,38; t = 2,4413;p < 0.05	
9e	Astrocyte Cre (N=7)	Number of XY Pairs<br>**Spearman r: 0.79**	
S1 d	Control (n = 6, N = 2)<br>AP5 (n = 6, N = 3)	**Friedman test**; p = 0.0008<br>Friedman statistic = 9.579<br>Dunn´s Multiple comparisons ///// Difference in rank sum<br>Before vs LFS; ´-7.50; p < 0.05<br>Before vs After; 4; ns<br>LFS vs After; ´9.50; p < 0.01	
S3 Control pathway	Control (n = 10; N = 6)<br>AP5 (n = 8; N = 6)<br>MCPG (n = 8, N = 5)<br>BAPTA (n = 9, N = 6)	**One-way ANOVA:**<br>F (3, 34) = 0.8, p = 0.53	
S4 LTP	Control (n = 8, N = 4)<br>BAPTA in astrocyte (n = 7, N = 4)	**Mann-Whitney U-test**<br>Control vs BAPTA, p = 0.27; z = 1.09	**Wilcoxon signed-rank test**<br>Control: p < 0.05; z = 2.48<br>BAPTA: p < 0.05; z = 2.15
S5b	Control (n = 8, N = 4)<br>TeTxLC (n = 7, N = 4)	**Mann-Whitney U-test**<br>Control vs TetxLC, p < 0.05; z = 3.00	
S7	ChR2 (n = 8, N = 4)<br>ChR2 + AP5 (n = 6, N = 4)	**One-way ANOVA:**<br>p < 0.01; t = 3.95	**Wilcoxon signed-rank test**<br>ChR2: p < 0.05; z = 2.48<br>ChR2 + AP5: p = 0.49; z = 0.68
s11	Vehicle (N = 6)<br>Neuronal Cre (N = 6)<br>Astrocyte Cre (N = 7)	**One-way ANOVA:**<br>p < 0.05; t = 8.45<br>Post-hoc Tukey Test´s test<br>Control vs AP5, p < 0.05; q = 3,675<br>Control vs SB 309580, p < 0.05; q = 3,375<br>SB 309580 vs AP5, p = 1; q = 0.3	

S13	Vehicle (n = 8; N = 5)<br>Neuronal Cre (n = 11, N = 6)<br>Astrocyte Cre (n =11, N = 7)	**One-way ANOVA:**<br>F (2, 29) = 11.634, p < 0.001<br>Post-hoc Bonferroni´s test<br>Vehicle vs Astrocyte, p < 0.05, z = 2.95<br>Vehicle vs Neuronal, p = 0.24, z = 1.39	**Wilcoxon signed-rank test**<br>Vehicle: p < 0.05; z = 2.48<br>Neuronal Cre: p < 0.01; z = 2.91<br>Astrocyte Cre: p = 0.11; z = 1.57
s14a	Vehicle (N = 11)<br>Neuronal Cre (N = 10)<br>Astrocyte Cre (N = 11)	Time: **Two-way ANOVA**: F (4, 116) = 79.78; p < 0.001<br>Infection: F (2, 116) = 0.8833; p = 0.42	
s14b	Vehicle (N = 11)<br>Neuronal Cre (N = 10)<br>Astrocyte Cre (N = 11)	**Kruskal-Wallis test**<br>Kruskal-Wallis statistic = 0,1854; p = 0,9114	
s14c	Vehicle (N = 7)<br>Neuronal Cre (N = 5)<br>Astrocyte Cre (N = 7)	**Two-way ANOVA:**<br>Source of Variation; p = 0.95<br>Interaction; p = 1<br>p < 0.0001	% of total variation<br>0,39<br>0,00<br>81,41
s14d	Vehicle (N = 7)<br>Neuronal Cre (N = 5)<br>Astrocyte Cre (N = 7)	**Two-way ANOVA**<br>Source of Variation<br>Interaction; p = 0.99<br>Factor; p = 1<br>Row Factor; p < 0.001	% of total variation<br>0,21<br>0,00<br>83,21
s14e	Vehicle (N = 7)<br>Neuronal Cre (N = 5)<br>Astrocyte Cre (N = 7)	**Two-way ANOVA:**<br>Source of Variation<br>Interaction; p = 0.56<br>Row Factor; p = 0.9<br>Time; p = 0.04<br>Subjects (matching) p < 0.0001	% of total variation<br>0,80<br>1,04<br>3,35<br>84,0259
s14f	Vehicle (N = 6)<br>Neuronal Cre (N = 6)<br>Astrocyte Cre (N = 7)	Time: **Two-way ANOVA**: F (4, 116) = 79.78; p < 0.001<br>Infection: F (2, 116) = 0.88; p = 0.42	
s15b	Vehicle (N = 7)<br>Neuronal Cre (N = 5)<br>Astrocyte Cre (N = 7)	**Kruskal-Wallis test**<br>Kruskal-Wallis statistic = 01,608; p = 0,4474	
s15c	Vehicle (N = 7)<br>Neuronal Cre (N = 5)<br>Astrocyte Cre (N = 7)	**Kruskal-Wallis test**<br>Kruskal-Wallis statistic = 1,662; p = 0,4356	
s15d	Vehicle (N = 7)<br>Neuronal Cre (N = 5)<br>Astrocyte Cre (N = 7)	**Kruskal-Wallis test**<br>Kruskal-Wallis statistic = 0,4091; p = 0,8150	
s15e	Vehicle (N = 7)<br>Neuronal Cre (N = 5)<br>Astrocyte Cre (N = 7)	**Two-way ANOVA**<br>Source of Variation<br>Interaction; p = 0.68<br>Factor; p = 0.43<br>Row Factor; p = 0.0013	% of total variation<br>1,65<br>3,71<br>26,91
s15f	Vehicle (N = 7)<br>Neuronal Cre (N = 5)<br>Astrocyte Cre (N = 7)	**Two-way ANOVA**<br>Source of Variation<br>Interaction; p = 0.15<br>Factor; p = 0.92<br>Row Factor; p < 0.0001	% of total variation<br>1,69<br>0,07<br>85,99

Supplementary Table. Summary of statistical details for all experiments, including number of animals and slices and statistical tests used.
