# Supplementary material for: Astrocytic p38α MAPK drives NMDA receptor-dependent long-term depression and modulates long-term memory
Source: Nat Commun. 2019 Jul 4;10:2968. doi: 10.1038/s41467-019-10830-9 (PMC6609681; doi:10.1038/s41467-019-10830-9)
Supplement: Supplementary file 3 — Reporting Summary [file 41467_2019_10830_MOESM3_ESM.pdf]

nature research

Corresponding author(s): Marta Navarrete & José A. Esteban

Reporting Summary

Nature Research wishes to improve the reproducibility of the work that we publish. This form provides structure for consistency and transparency in reporting. For further information on Nature Research policies, see Authors & Referees and the Editorial Policy Checklist.

Statistical parameters

When statistical analyses are reported, confirm that the following items are present in the relevant location (e.g. figure legend, table legend, main text, or Methods section).

n/a	Confirmed	
☐	☒	The exact sample size (n) for each experimental group/condition, given as a discrete number and unit of measurement
☐	☒	An indication of whether measurements were taken from distinct samples or whether the same sample was measured repeatedly
☐	☒	The statistical test(s) used AND whether they are one- or two-sided<br>*Only common tests should be described solely by name; describe more complex techniques in the Methods section.*
☐	☒	A description of all covariates tested
☐	☒	A description of any assumptions or corrections, such as tests of normality and adjustment for multiple comparisons
☐	☒	A full description of the statistics including central tendency (e.g. means) or other basic estimates (e.g. regression coefficient) AND variation (e.g. standard deviation) or associated estimates of uncertainty (e.g. confidence intervals)
☐	☒	For null hypothesis testing, the test statistic (e.g. F, t, r) with confidence intervals, effect sizes, degrees of freedom and P value noted<br>*Give P values as exact values whenever suitable.*
☒	☐	For Bayesian analysis, information on the choice of priors and Markov chain Monte Carlo settings
☒	☐	For hierarchical and complex designs, identification of the appropriate level for tests and full reporting of outcomes
☒	☐	Estimates of effect sizes (e.g. Cohen's d, Pearson's r), indicating how they were calculated
☐	☒	Clearly defined error bars<br>*State explicitly what error bars represent (e.g. SD, SE, CI)*

Our web collection on statistics for biologists may be useful.

Software and code

Policy information about availability of computer code

Data collection	Electrophysiological data was collected using Multiclamp 700A/B amplifiers and pClamp software (Molecular Devices).<br>Behavioral data was collected with Anymaze (Stoelting) video tracking system.<br>Imaging data was colleted with Zeiss Zen software
Data analysis	Data collected was further analyzed with GraphPad Prism 7 and/or SigmaPlot 11.00. Images were analyzed with ImageJ (public domain software developed at the US National Institutes of Health) and Matlab2016. Electrophysiology data were analyzad with Clampfit routine of pClamp 10.7.0

For manuscripts utilizing custom algorithms or software that are central to the research but not yet described in published literature, software must be made available to editors/reviewers upon request. We strongly encourage code deposition in a community repository (e.g. GitHub). See the Nature Research guidelines for submitting code & software for further information.

Data

Policy information about availability of data

All manuscripts must include a data availability statement. This statement should provide the following information, where applicable:

- Accession codes, unique identifiers, or web links for publicly available datasets
- A list of figures that have associated raw data
- A description of any restrictions on data availability

The data supporting these findings are available from the corresponding author upon request.

Field-specific reporting

Please select the best fit for your research. If you are not sure, read the appropriate sections before making your selection.

☒ Life sciences ☐ Behavioural & social sciences ☐ Ecological, evolutionary & environmental sciences

For a reference copy of the document with all sections, see nature.com/authors/policies/ReportingSummary-flat.pdf

Life sciences study design

All studies must disclose on these points even when the disclosure is negative.

Sample size	We did not perform power analysis to predetermine sample sizes, but our sample sizes are similar to those generally employed in the field.
Data exclusions	For all experiments, the animals' brain were processed for histology to confirm virus infection sites. Data will be excluded if animals display an inaccurate viral injection site.<br>Electrophysiology recordings were considered when the series and input resistances, resting membrane potential and stimulus artefact duration did not change > 20%.
Replication	The experimental findings were reliably reproduced. Detailed methods and description of protocols of the experiments will allow for easy reproduction.
Randomization	For all behavior tests, littermates were randomly coded and allocated into experimental and control groups.
Blinding	All behavioral assays were carried out in a blind manner, so that the experimenter and the person carrying out the analyses did not know the genotype or the treatment that the animal had received. Collection and analysis of electrophysiological data were performed blind to the condition of the experiments.

Reporting for specific materials, systems and methods

Materials & experimental systems

n/a	Involved in the study	
☒	☐	Unique biological materials
☐	☒	Antibodies
☐	☒	Eukaryotic cell lines
☒	☐	Palaeontology
☐	☒	Animals and other organisms
☒	☐	Human research participants

Methods

n/a	Involved in the study	
☒	☐	ChIP-seq
☒	☐	Flow cytometry
☒	☐	MRI-based neuroimaging

Antibodies

Antibodies used	anti-p38alpha MAPK (Santacruz, sc-535)<br>anti-p38beta MAPK (Thermo Scientific, 33-8700)<br>anti-actin (Millipore, MAB1501R)<br>anti-VAMP3 Thermo Scientific, PA1-767A)<br>anti-Synaptobrevin (Synaptic Systems, clone 91-1)<br>anti-MAP2 (Covance, PCK-554P)<br>anti-GFAP (Sigma, G3893)<br>anti-Neu-N (Abcam,ab177487)

	anti-GFP (Roche, 11814460001)<br>anti-Iba1 (Wako, 019-19741)<br>peroxidase-conjugated anti-rabbit (Jackson ImmunoResearch)<br>peroxidase-conjugated anti-mouse (Jackson ImmunoResearch)<br>anti-rabbit Alexa 647 (Thermo Fisher, A-31573)<br>anti-chicken Alexa 488 (Thermo Fisher, A-11039)<br>anti-mouse Alexa 488 (Thermo Fisher, A-21121)<br>anti-mouse Alexa 555 (Thermo Fisher, A-21137)
Validation	All antibodies are validated for species by manufacturer.

Eukaryotic cell lines

Policy information about cell lines

Cell line source(s)	HEK293-FT.
Authentication	The cells were authenticated by the vendor and no further authentication was performed in the laboratory.
Mycoplasma contamination	Cell lines were tested negative for mycoplasma contamination using a colorimetric detection system.
Commonly misidentified lines<br>(See ICLAC register)	None of the cell lines used is listed as commonly misidentified.

Animals and other organisms

Policy information about studies involving animals; ARRIVE guidelines recommended for reporting animal research

Laboratory animals	C57BL/6; p38alox/lox; p38alpha-/-; p38beta-/- and p38alpha(CaMKIIa)-/-, IP3R2-/- mice<br>Wistar rats. Animals of both genders were used.
Wild animals	The study did not involve wild animals.
Field-collected samples	No field-collected samples were used.
